# Room temperature texturing of austenite/ferrite steel by electropulsing

**DOI:** 10.1038/srep42732

**Published:** 2017-02-14

**Authors:** Alireza Rahnama, Rongshan Qin

**Affiliations:** 1 International Digital Laboratory, Warwick Manufacturing Group, University of Warwick, Coventry CV4 7AL, United Kingdom; 2 School of Engineering and Innovation, The Open University, Walton Hall, Milton Keynes MK7 6AA, United Kingdom

## Abstract

The work reports an experimental observation on crystal rotation in a duplex (austenite + ferrite) steel induced by the electropulsing treatment at ambient temperature, while the temperature rising due to ohmic heating in the treatment was negligible. The results demonstrate that electric current pulses are able to dissolve the initial material’s texture that has been formed in prior thermomechanical processing and to produce an alternative texture. The results were explained in terms of the instability of an interface under perturbation during pulsed electromigation.

Electropulsing treatments, an instantaneous high energy input, has characteristics such as fast heating, electromagnetic force, high speed impact and reduced thermodynamic potentials^1^,^2^,^3^,^4^. In an elegant study, it was shown that current pulses promote structural evolution towards a state with lower electrical resistance^5^. When electric current pulses are imposed on a material, atoms can be rearranged to a structure with minimum electrical resistance^6^,^7^,^8^. The application of electropulsing has been extensively studied in recent years. These studies showed that the electropulsing treatment can have a marvellous application foreground in affecting the plasticity^9^,^10^,^11^,^12^, recrystallization^12^,^13^,^14^,^15^, phase transformation^16^,^17^,^18^,^19^,^20^, microstructural evolution^1^,^2^,^21^,^22^, cast microstructure^23^ and fatigue life^24^ of alloys. The effect of electropulsing treatment on the formability of metallic materials were also studied extensively^25^,^26^,^27^. It was shown that the microstructure and texture can be significantly affected by electroplastic rolling. Moreover, it was reported that electric current pulses is able to change the recrystallization texture by affecting cube and shear band nucleation^28^. In other studies, it was reported that electropulsing affects the grain orientation in cold-rolled 3% Si steel because recrystallized nuclei formed in a preferential direction along the current direction during the primary period of recrystallization^29^,^30^,^31^. It was also found that electric current direction significantly alters the microstructural evolution in a Cu-Zn binary phase (*α* + *β*) alloy^32^. However, the effects of electric current pulses on the crystal rotation at ambient temperature with negligible temperature rise during the treatment have not been reported in the literature.

The present work reports the crystal rotation in a duplex (austenite (FCC) + ferrite (BCC)) steel. The treatment was performed at room temperature, and it was discovered that the initial texture due to the prior thermomechanical processing was wiped away and a new texture was formed by application of electric current pulses to the material. The temperature rising due to Ohmic heating was negligible (<5 °C). This research indicates a potential to use electropulse as an add-on process to texturize the materials. The results are explained in terms of the instability of the interface as a result of electric current flow.

## Experiment

### Materials and electropulsing

The material was prepared via the conventional ingot-making metallurgical routine and the chemical compositions in weight percentage were confirmed to be 0.15 C, 1.6 Si, 2 Mn, 3 Al, 0.1 Cr. The ingot was rolled at 800 °C to a sheet with 2.64 mm thickness and then chilled. The sheet was cut into 30 mm × 3.42 mm × 2.64 mm samples and grouped randomly for subsequent electropulsing treatment.

The electropulse was generated by an Avtech AV-108F-B-P pulse generator which converted the direct current into pulses. The direct current power source has an output power of 80 watts and output electric potential of 20 volts. The pulse width, peak current intensity, pulse frequency and pulse trigger mode are programmable. The testing steel sample was connected to two copper electrodes from both ends to form a current circuit. No internal stress might occur if there was a current-induced temperature rising because both sample and electrodes were hold freely rather than fixed to certain positions. An oscilloscope was connected to the circuit to monitor the electropulse signal. All the pulses applied in this work were chosen to have 20 *μ*s pulse width and 1.018 × 10^7^ A/m^2^ peak current density. The multiple pulses are applied at a frequency of 1 pulse per second. Throughout the experiments, the temperature of the samples were measured by an attached thermocouple. 50, 100 and 1000 electric pulses were applied to the material.

### Microstructural characterisation

The microstructural characterisations were performed by LEO Gemini 1525 high resolution field emission gun scanning electron microscopy (FEG-SEM) and electron backscatter diffraction microscopy (EBSD). The samples for scanning electron microscopy observations were prepared by the conventional method using diamond pastes and etched in 2% nital etching solution. A specific rectangular region in the middle of samples were indented after polishing through a microhardness tester in order to obtain EBSD maps and inverse pole figures (IPF) for both FCC and BCC structure from the exactly same region after each electropulsing treatment. In this way, we were able to study the orientation distribution of the grains in a semi-*in-situ* way.

## Results and Discussion

**Figure 1:**
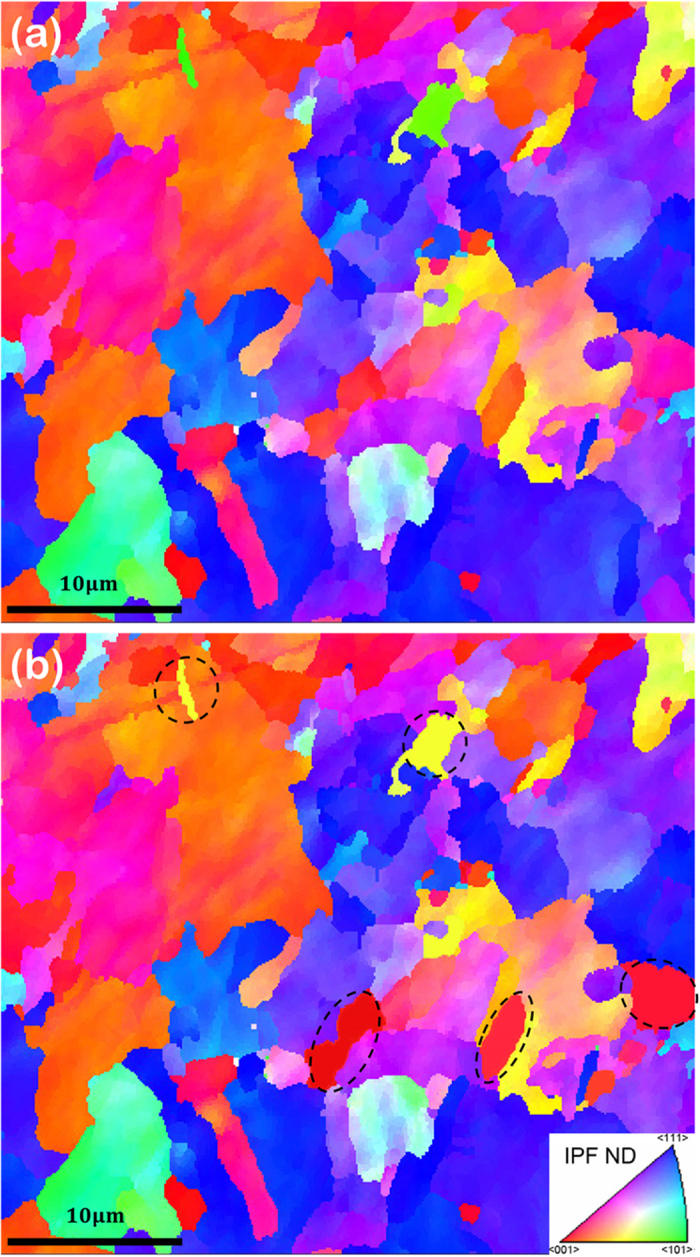
EBSD maps showing the orientation according to the surface normal (ND) (**a**) before electropulsing and (**b**) after 50 electric current pulses. (The colour code is presented according to the associated standard IPF.)

**Figure 2:**
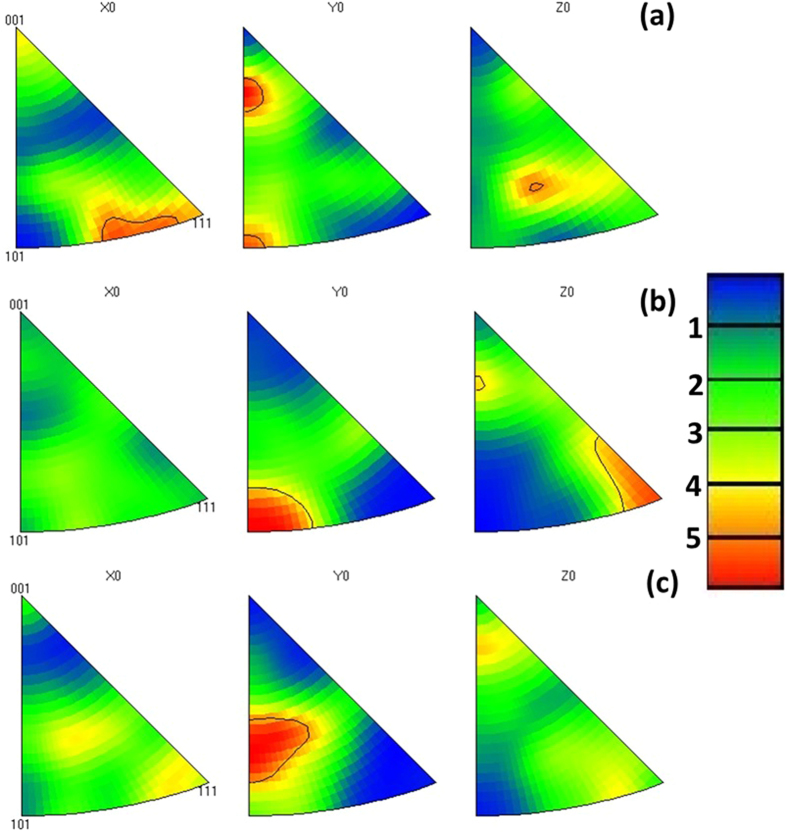
Pole figures of the BCC phase for (**a**) without electropulsing treatment, (**b**) after 100 pulses and (**c**) after 1000 pulses.

Figure 1 shows the orientation of the grains relative to the surface normal direction (ND) for the same zone (a) before and (b) after imposing 50 electric current pulses with a frequency of 1 Hz. As can be seen in this figure, the colour codes of a few grains changed (marked by dashed ellipsoid) after the treatment indicating that those grains rotated and obtained new orientations. Figure 2a shows the IPF of the sample before electropulsing. The map shows a specific texture because of prior thermomechanical processes. After applying 100 pulses, the poles in *x*_0_ faded away while a strong pole formed in 〈101〉 direction in *y*_0_ and one in 〈111〉 direction in *z*_0_, as can be seen in Fig. 2b. By applying more electric current pulses (1000 pulses), new poles formed in *x*_0_ along the 〈111〉 direction while the pole in *y*_0_ rotated towards 〈001〉 direction compared to that after 100 pulses. The poles in *z*_0_ also disappeared although it still showed some texture close to 〈001〉 direction, as can be seen in Fig. 2c.

**Figure 3:**
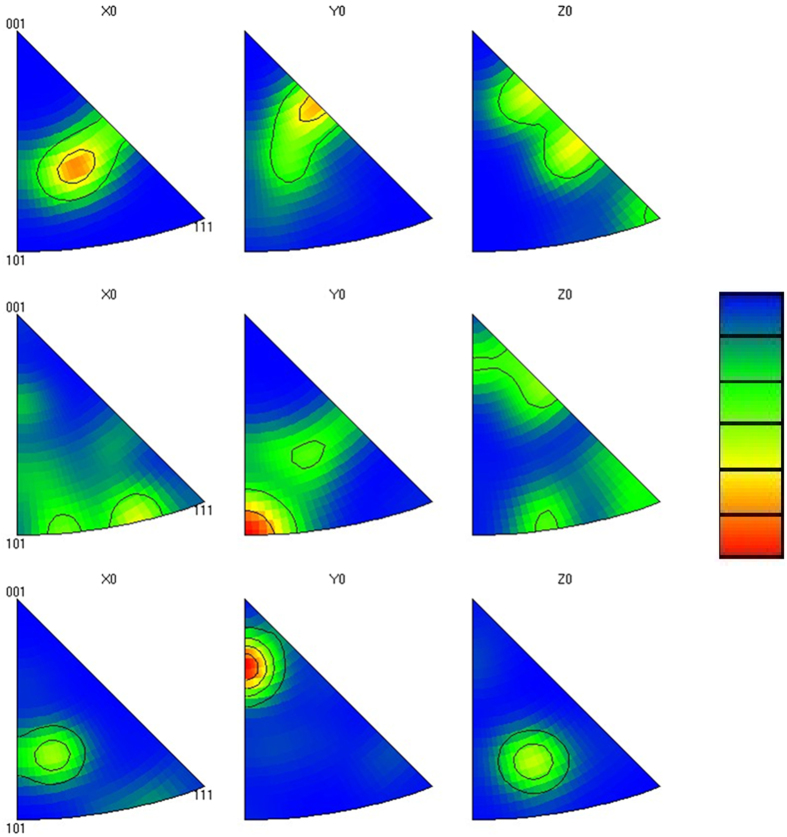
Pole figures of the FCC phase for (**a**) without electropulsing treatment, (**b**) after 100 pulses and (**c**) after 1000 pulses.

Figure 3a shows the IPF for the FCC structure before the electropulsing treatment. Again, it shows a texture because of the prior thermomechanical processing. By applying 100 current pulses, the *x*_0_ poles almost disappeared while a strong pole formed in *y*_0_ along the 〈101〉 direction. *z*_0_ poles also dissolved after imposing 100 pulses, as shown in Fig. 3b. Increasing the number of electric pulses to 1000 (Fig. 3c), a new pole close to 〈101〉 direction formed in *x*_0_, a strong pole was generated in *y*_0_ near 〈001〉 and a pole appeared in *z*_0_ close to 〈101〉 direction in *z*_0_. These observations, both in BCC and FCC structure, confirmed a crystal rotation towards certain directions which are believed to possess lower resistance to electric current flow, for example electrical resistance of 〈001〉 in FCC structure is lower than of 〈111〉 direction^1^.

**Figure 4:**
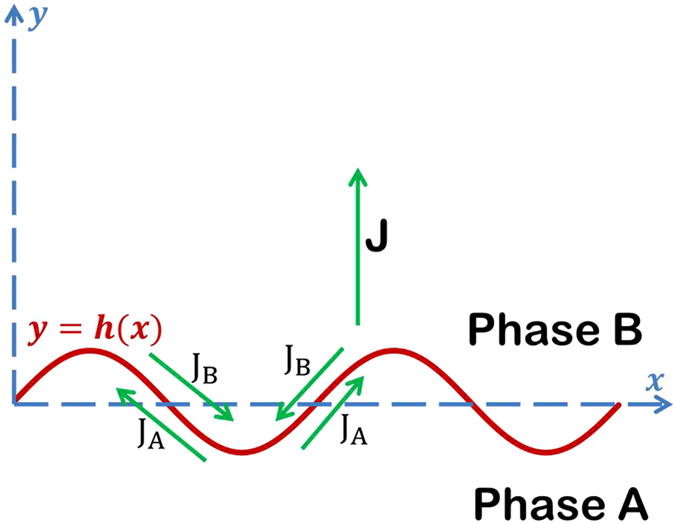
Interface instability caused by interface diffusion during the electropulsing treatment.

Here, we employ the electric-current-induced interface instability theory which was developed by Srolovitz *et. al*.^33^ to explain our experimental observations. According to the theory, the diffusion flux along the interface is affected by three causes: firstly chemical potentials change due to electric field (*ϕ*), secondly the associated internal tensile or compressive stresses which are created due to the accumulation or depletion of matter at the interface (*σ*) and finally the curvature of the interface (*k*). These three causes can be summarized in the following mathematical formula:


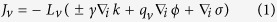


where *γ* refers to interface tension. *L*_*v*_ is the mobility and *q*_*v*_ is the volume charge of *v*-th component. If the interface is planar (y = 0), the migration and thus the rotation of grains is impossible as the flux is perpendicular to the interface. Thus, the interface is stable. But if we assume that there is perturbations at the interface (y = h(x)), as shown in Fig. 4, the flux *J* has components along the interface. These components promote the movement of the interface and thus make the crystal rotation possible even at room temperature. This divergence of diffusion flux will lead to the shift of the interface as follows:


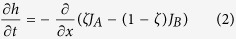


*ζ* depends on the elastic modulus of the phases. There are two reasons for the instability of the interface during the electropulsing: first the difference in atomic charges of constituent elements and secondly the difference in the mobilities of atoms in difference phase and grains. Because the constituent elements of the current materials have different charges and mobilities in austenite from those in ferrite, the interfaces became unstable during the treatment and made the crystal rotation and the shift of grain boundaries possible at room temperature. This theory, thus, explains the observed phenomenon and shows that crystal rotation is possible at room temperature without any increase in the temperature of the material.

However, there should be a critical electric current density to drive the crystals to start the rotation. The crystal rotation requires overcoming a resistive force. Only when the electric current density is larger than the critical value, the electropulse is able to overcome the resistive force and to create the instability. This is similar to that of the critical electric current to initiate the electroplasticity.

In summary, we have observed crystal rotation during the electropulsing treatment in a duplex (austenite + ferrite) steel at room temperature. The grains were rotated towards certain directions which are believed to possess lower electrical resistance. The treatment was not accompanied with considerable temperature rise in the material. Numerical calculation shows that the maximum temperature rising due to ohmic heating is 0.217 K/s. This is unlikely to raise samples’ temperature due to heat dissipation into the environments. However, it needs to be noted that current density as well as frequency are determining factors that can be the subject of future studies in order to come to a general conclusion regarding the effect of electric current pulses on the texture evolution of materials at ambient temperature with no increase in the temperature of materials or in the operation temperature. The presented study reveals a potential of using electropulsing treatment for the texturizing of materials. Electropulsed-induced crystal rotation at ambient temperature means that the treatments can be very efficient in terms of cost and energy consumption.

## Additional Information

**How to cite this article:** Rahnama, A. and Qin, R. Room temperature texturing of austenite/ferrite steel by electropulsing. *Sci. Rep.* **7**, 42732; doi: 10.1038/srep42732 (2017).

**Publisher's note:** Springer Nature remains neutral with regard to jurisdictional claims in published maps and institutional affiliations.
